# Investigating the Role of Goals and Motivation on Waste Separation Behavior Through the Lens of the Theory of Reasoned Goal Pursuit

**DOI:** 10.1007/s00267-023-01820-1

**Published:** 2023-05-03

**Authors:** Alessandro Concari, Gerjo Kok, Pim Martens, Nathalie Brink

**Affiliations:** 1 University College Venlo, Faculty of Science and Engineering, Maastricht University, Maastricht, The Netherlands; 2 Department of Work & Social Psychology, Faculty of Psychology and Neuroscience, Maastricht University, Maastricht, The Netherlands; 3 Maastricht Sustainability Institute, Maastricht University, Maastricht, The Netherlands

**Keywords:** Waste separation behavior, Recycling, Theory of planned behavior, Theory of reasoned goal pursuit, Goal systems theory, Pro-environmental behavior

## Abstract

Nowadays the prediction and change of waste-related behaviors represent a key topic for scholars and policy makers. The theoretical mainstays applied to waste separation behavior, such as the Theory of Planned Behavior (TPB), the Norm Activation Model and the Value Belief Norm, do not include the construct of goal in their formulation. Other goal-focused theories, such as the Goal Systems Theory (GST), lack applications on separation behavior. Recently, Ajzen and Kruglanski (2019) have proposed the Theory of Reasoned Goal Pursuit (TRGP) which combines TPB and GST. Considering TRGP has the potential to offer further insights on human behavior and, to our knowledge, there is no application of TRGP to recycling behavior yet, this paper analyses waste separation behavior of households in Maastricht and Zwolle (The Netherlands) under the lens of TRGP. Although waste separation behavior represents a kind of habitual behavior, this paper highlights the influence of goals and motivation on intention to separate waste. Furthermore, it offers some indications to promote behavior change and some suggestions for future research directions.

The analysis of the academic papers on pro-environmental behavior in the last years indicates that waste recycling behavior remains topical. The prediction and change of waste-related behaviors, like separation, reduction, re-utilization, represent a key topic for scholars, scientist, politicians, waste service providers and policymakers. Some researchers are more focused on the reasoned part of human behavior like intention, attitude, norms, awareness of consequences, ascription of responsibility, personal values, beliefs (McCarty and Shrum 2001); whereas others highlight the importance of less volitional predictors of waste-related behaviors like habits (Cheung et al. 1999; Lavelle et al. 2015) or emotions (Carrus et al. 2008). Contextually, we recognize that, in the specific field of waste-related behavior, the constructs of goal and motivation are actually not sufficiently addressed in combination with the typical precursors of behavior like intention and norms. Looking at the theoretical mainstays applied to recycling behavior, the Theory of Planned Behavior (TPB; Ajzen 1991) and its predecessor, the Theory of Reasoned Action (TRA; Ajzen and Fishbein 1970), the Norm Activation Model (NAM; Schwartz 1977) and the Value Belief Norm (VBN) Theory (Stern 2000) do not include the construct of “goal” in their formulation. Although TPB has proved to be a robust framework to explain pro-environmental behaviors at different levels (e.g., managerial, household, consumer) (Li et al. 2019; Miafodzyeva and Brandt 2013), Perugini and Bagozzi (2001) move a step forward by adding desire and anticipated emotions to the TPB framework. Certainly, human behavior is goal-driven as well, and several theories have offered frameworks to give the right emphasis to this important precursor of behavior, for example the Goal Setting Theory (Latham and Locke 1979) and the Goal Systems Theory (GST; Kruglanski et al. 2015; Kruglanski et al. 2002). Recently, Ajzen and Kruglanski (2019) have proposed the Theory of Reasoned Goal Pursuit (TRGP) which combines TPB and GST. The TRGP has the potential to offer further insights on human behavior and as, to our knowledge, there is no application of TRGP to recycling behavior yet, this paper analyses waste separation behavior of the households in Maastricht and Zwolle (The Netherlands) under the lens of TRGP. Acknowledging that TPB has been successfully tested in different contexts (Carmi et al. 2015), this paper aims at both understanding the effect of the inclusion of the goals and motivation among the TPB precursors, and offering valid suggestions to policy makers and service providers in the definition of effective waste management measures at the household level. Although waste separation behavior represents a kind of habitual behavior, driven by a consolidated waste management procedure in many advanced economies, we expect not only to corroborate the validity of the typical TPB constructs in explaining waste sorting behavior, but also to verify the enhanced predictive capability of TRGP for this type of habitual behavior.

## Literature Review and Theoretical Framework

The TPB (Ajzen 1991) represents the most widespread framework for analyzing recycling behavior (Yuriev et al. 2020). This theory can be considered the evolution of the TRA (Ajzen and Fishbein 1970); both of them analyze and predict social behavior through “a set of hierarchically linked constructs” (Barr 2004, p. 233). Intention is the immediate predictor of behavior, whereas attitude, subjective norms and perceived behavioral control (PBC) (the latter in the case of TPB only) are the precursors of intention. Intention “represents the person’s motivation in the sense of his or her conscious plan to exert effort to carry out a behavior” (Eagly and Chaiken 1993, p. 168). It is influenced, in turn, by attitude, which measures “the degree to which a person has a favorable or unfavorable evaluation or appraisal of the behavior in question” (Ajzen 1991, p. 188). Fishbein and Ajzen (2011) highlight the evaluative and bipolar nature of attitude; in fact, they ascribe “to individuals a position on a unitary evaluative dimension with respect to an object, a dimension that ranges from negative to positive through a neutral point” (Fishbein and Ajzen, 2011, p. 76). Numerous studies have proven the direct relationship between attitude and intention; in fact, a positive attitude toward the behavior reinforces the intention to perform the behavior. Among the precursors of intention, attitude often represents a very influential one (Aboelmaged 2021; Khan et al. 2020; Ling et al. 2018; Seng et al. 2021; Wang et al. 2019; Zhang et al. 2021). Another precursor is subjective norms which represent “the perceived social pressure to perform or not to perform the behavior” (Ajzen 1991, p. 188). In fact, this construct measures the influence of the society or important others (e.g., parents, partner) on the individual; Fishbein and Ajzen (2011) use the “term subjective norm because this perception may or may not reflect what most important others actually think should be done” (p. 131). In relation to recycling behavior, several studies have confirmed the importance of norms in predicting intention and behavior, however at a lower level compared to attitude both at the individual level (Botetzagias et al. 2015; Wang et al. 2020) and at the organizational level (Khan et al. 2020). The third precursor of intention, PBC, measures “the perceived ease or difficulty of performing the behavior” (Ajzen 1991, p. 188). This construct considers both the capacity and the autonomy of the individual of performing the behavior. Furthermore, Ajzen and Fishbein consider the direct and unmediated effect of PBC on behavior. An overall analysis of this construct in scientific papers indicates that the level of influence of PBC on recycling intention and behavior is significant, even though we may notice different levels of significance among case studies (Liao and Li 2019; Nigbur et al. 2010; Wang et al. 2020).

Although the original TPB framework has been successfully applied to recycling behavior, numerous scholars have added constructs to make it fit better to specific situations, for example, past behavior, emotions, habits and desire. In particular, the model of goal directed behavior (MGB) (Perugini & Bagozzi, 2001) enriches TPB by adding positive and negative anticipated emotions, past behavior (frequency) and desire to the typical TPB constructs.

In MGB the immediate predictor of intention is desire which “mediates the effects of attitude, subjective norms, PBC and anticipated emotions on intention and behavior” (Parkinson et al. 2018, p. 840); at the same time, PBC does not directly influence intention but desire and behavior. Considering some scholars criticize TPB for not explaining “how intentions become energized” (Perugini and Bagozzi 2001, p. 83), Perugini and Bagozzi (2001) introduce desire as “the motivational impetus for intention” (p. 83); in turn, attitude, subjective norms and PBC are the catalysts to fire up the dormant desire. Another important additional construct is anticipated emotions which are the referents of personal goals; in fact, Perugini and Bagozzi (2001, p. 83) state that “anticipated emotions function as independent variables based upon a decision process that takes into account judged consequences of goal achievement and goal failure”. Furthermore, Perugini and Bagozzi (2001) consider (frequency of) past behavior as a predictor of desire, intention and behavior; on the contrary Ajzen infers that the residual effects of past behavior are mediated by PBC. In relation to the application of MGB to recycling behavior, Carrus et al. (2008) find a consistent relationship between negative anticipated emotions and desire to recycle; furthermore, this relationship is more statistically significant than the one between attitude and desire, or PBC and desire.

Building on the fact that “most behaviors are functional to goal achievement and can be better understood and predicted by considering relevant constructs at the goal level” (Perugini and Conner 2000, p. 705), Perugini and Conner (2000) create the extended MGB (EMGB) framework by integrating MGB with goal desires and goal perceived feasibility (GPF). The former is an antecedent of behavioral desire and measures “the valence of an action’s end state” (Liberman and Trope 1998, p. 7), the latter is an antecedent of PBC and measures how easy or difficult it is to reach the end state (Liberman and Trope 1998). Another peculiarity of EMGB is the replacement of the construct of intention with the broader one of volition, which measures different aspects such as intention, commitment, effort and planning. Unfortunately, to our knowledge, EMGB has not yet been applied to recycling behavior but to different sectors such as tourism and hospitality (Kim and Preis 2016).

At present, the suitability of TPB for fully understanding and predicting pro-environmental behavior is still undergoing numerous tests. In this regard, it is worth recalling Staats (2003) who concludes that

“the [TPB] model will perform best when the behavior under consideration is very reasoned, or very planned. That is, the more attention is given to consciously considering all the relevant factors (behavioral, normative and control beliefs) the better will be the prediction” (p. 185).

Consequently, Staats distinguishes between the application of TPB to important decisions with long-term and irreversible effects, versus behaviors with less stable relationships among components (e.g., attitude and related beliefs).

Another widespread framework for analyzing recycling behavior is the Norm Activation Model (NAM) and the Value Belief Norm (VBN). NAM has been proposed by Schwartz (1977) assuming that the activation of personal norms strongly influences human behavior; in turn, personal norms are activated by awareness of consequences (AC) and ascription of responsibility (AR). The VBN by Stern (2000) builds on NAM by integrating the concepts of biospheric, altruistic and egoistic values. Starting from that, VBN defines “a causal chain of five variables leading to behavior: personal values (especially altruistic ones), NEP (New Environmental Paradigm), AC and AR beliefs … and personal norms for proenvironmental action” (Stern 2000, p. 412). Whereas TPB is a generic conceptual framework applied to very different contexts, VBN offers a framework for environmentally significant individual behavior. In fact, VBN, building on the concepts of environmentalism, namely the “propensity to take actions with proenvironmental intent” (Stern 2000, p. 411), and environmentally significant behavior, highlights the impact of individual behavior on environment and its responsibility for minimizing this impact.

Some authors have also investigated the reasons for selecting a specific framework. It is worth recalling Bamberg and Moser (2007) who explain the selection of a reasoned choice approach (TRA or TPB) with researchers’ need to focus on the individual’s self-interests. On the contrary, scholars more interested in pro-social behavior opt for a framework based on NAM or VBN.

The mentioned meta-analysis and systematic literature reviews (Concari et al. 2020; Concari et al. 2022; Li et al. 2019; Miafodzyeva and Brandt 2013) also show that goal constructs have never actually been fully and directly considered in the study of recycling behavior. First, it is worth recalling some theoretical frameworks focusing on goals. Latham and Locke (1979)’s “Goal Setting Theory” focuses on the individual setting their personal goals to satisfy their personal needs. So far, this theory lacks applications to household behavior.

The GST (Kruglanski et al. 2002) considers goals as the motivators of action, being “a mental representation whose contents are of motivational significance” (Kruglanski 1996, p. 599). Given that goals are dynamic and can be reached in different ways, Kruglanski et al. (2015) focus on the means to reach these goals as well, and define the concept of multifinality, equifinality and counterfinality. In fact, a goal can be reached by one or more means, and, vice versa, one single mean can satisfy one or more goals; furthermore, a goal can represent a top priority in our life or can compete with other objectives at other times. Kruglanski (1996) considers goals as “a desirable future state of affairs one intends to attain through action” (p. 600); in fact, goals are defined in terms of desirability, attainability and accessibility. Consequently, “only contextually available means can be considered for selection, and among these available means the most salient, vivid, and accessible will win out” (Bargh et al. 2010, p. 280). Overall, the GST postulates that goal systems have motivational and cognitive properties (Kruglanski et al. 2002). The former are driven by the “principle of subjective utility, which determines goal-commitment and mean choice” (Kruglanski et al. 2002, p. 342); moreover, the strive for a goal is influenced by persistence of pursuit and affective feedback. The latter are characterized by structural and allocational properties, namely the type of links between goals and means (interconnectedness), and the mental resources availability in a “constant sum” game. Being that the cognitive properties often take over the motivational ones, goals may range from short term and narrow objectives to long term ambitions (Kruglanski et al. 2002). Unfortunately, in the academic literature there is a very limited number of papers applying this theoretical framework to pro-environmental behavior, in particular to recycling behavior. Nielsen (2017) proposes a theoretical analysis of environmental behaviors in terms of goal setting and striving; Devezer et al. (2014) analyze the effect of goal failure and the importance on environmental friendly behaviors; Corrégé et al. (2018) study the effect of priming goals through social norms to improve energy-efficient behavior.

Quite recently Ajzen and Kruglanski (2019) combine TPB and GST into the TRGP to expand the predictive capabilities of their respective original frameworks: “whereas the TPB is a bottom-up approach that centers on the behavior as a point of reference, the GST represents a top-down approach in which the goals drive (and hence explain) the behavior undertaken in their service” (Ajzen and Kruglanski 2019, p. 777). In this regard, TRGP represents a novel approach; therefore, it is worth understanding the possible weaknesses of TPB as well as analyzing the constructs of goals and motivation, their roles and how they are connected to the TPB constructs.

Acknowledging the great utility of TPB in analyzing different types of behavior, Ajzen and Kruglanski (2019) concur that “the TPB’s behavior focus omits an important consideration, namely that behaviors are usually performed in the service of certain goals” (p. 777). In this regard, Ajzen and Kruglanski (2019) admit that, in numerous applications of TPB, the sample under investigation is under the (explicit or implicit) influence of active goals; for example, studies on people trying to lose weight are likely to involve individuals having the goal of being healthy or good-looking. At the same time, other behaviors may be driven by less evident or fluctuating goals, therefore their analysis through the lens of TPB may reveal some limitations in its explanatory power. The key role of goals in TRGP builds on the fact that attitude and subjective norms (which are considered “the central motivators of intention and behavior in the TPB” by Ajzen and Kruglanski (2019), p. 777) may not be enough to justify the initiation of a behavior until the individual thinks that the specific behavior is a mean to achieve an active goal. For example, an individual may show a positive attitude toward performing physical activity and may feel the social pressure of regularly executing this activity, but, until the individual realizes that regularly exercising is a mean to lose weight, he/she may not develop the intention to perform physical activity (nor starting that activity).

In the formulation of TRGP, Ajzen and Kruglanski (2019) posit that a behavior is driven by two types of goals (which may support each other in some situations or may contrast each other at other times): active procurement goals (APG) and active approval goals (AAG). The former goals aim at achieving the desired outcomes and experiences coming from the execution of a specific behavior, whereas the latter aim at obtaining the approval of important reference people.

Moreover, Ajzen and Kruglanski (2019) highlight that a strong intention to execute a behavior is also based on “a high level of motivation, that is a strong desire to perform the behavior” (p. 775); therefore, they assign to motivation the role of immediate predictor of intention. In turn, “motivation to initiate a contemplated behavior depends first and foremost on the perceived likelihood or expectancy that performing that behavior will bring about desired goals, as well as on the subjective values or magnitude of these goals” (Ajzen and Kruglanski 2019, p. 775).

Another key point of TRGP is the activation of goals; in fact, only active goals trigger (in the individual’s mind) the analysis of behavioral options to achieve those goals; conversely, if the goals are not active, the behavioral options do not become relevant. The activation of a procurement goal makes the individual consider the possibility of achieving this goal; in our case, if an individual deems environmental protection very important for his/her daily life, his/her attitude towards recycling becomes relevant. Similarly, AAGs indicate the personal aim at gaining the approval of important others; in this case study, if individuals consider recycling as an important way to get the approval of significant social referents, their subjective norms become relevant.

In the TRGP formulation, the concept of PBC does not substantially change. Ajzen and Kruglanski (2019) connect the concept of PBC with goals by underlining that the achievement of APGs and AAGs is obviously related to the ability of executing the behavior as well. In TRGP, PBC moderates the effect of motivation on intention, whereas actual behavioral control (ABC) moderates the relationship intention-behavior.

**Fig. 1:**
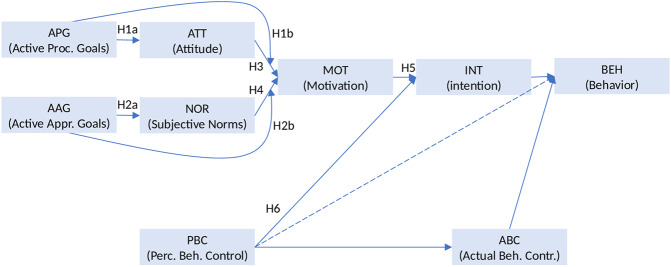
Research framework – Hypotheses testing

In line with the TRGP, our hypotheses are as follows (see Fig. 1):

H1a_APG-ATT_: Active procurement goal (APG) positively affects attitude (ATT)

H1b_APG-MOT_: Active procurement goal (APG) positively affects motivation (MOT)

H2a_AAG-NOR_: Active approval goal (AAG) positively affects subjective norms (NOR)

H2b_AAG-MOT_: Active approval goal (AAG) positively affects motivation (MOT)

H3_ATT-MOT_: Attitude (ATT) positively affects motivation (MOT)

H4_NOR-MOT_: Subjective norms (NOR) positively affect motivation (MOT)

H5_MOT-INT_: Motivation positively affects waste sorting intention (INT)

H6_PBC-INT_: PBC positively affects waste sorting intention (INT)

## Methodology

### Research Design

This paper is based on a research process made of four subsequential steps (Zhang et al. 2021). First, it reviews the applicable literature in order to analyze the theoretical frameworks applied to separation behavior and the related constructs; then it proposes the hypotheses testing the TRGP (previous section). Second, it defines a questionnaire (based on these hypotheses) through an initial eliciting questionnaire, followed by the pre-test on a limited sample, and finally the distribution of the survey with an adequate sample size (N) (this section). Third, it applies Structural Equation Modeling (SEM) via a two-stage procedure (Anderson and Gerbing 1988; Morrison et al. 2017): initial assessment of the measurement model through Confirmatory Factor Analysis (CFA), subsequent assessment of both measurement and structural models, and hypothesis testing (Results section). Fourth, it discusses the results (Discussion section), it proposes some suggestions for interventionists and future research, and it highlights main limitations (Conclusions section).

In relation to the second step, this study applies a quantitative research method to investigate the application of the TRGP to waste sorting behavior (Strydom 2018) in Maastricht and Zwolle (The Netherlands). The data were collected between March and July 2020. Considering that the research took place during COVID-19 pandemic, the questionnaires were distributed on-line.

### Structure of Questionnaire, Constructs and Measures

The questionnaire is made of different parts analyzing socio-demographical aspects (gender, education, employment status, type of dwelling, age range, number of people living in the household), socio-psychological factors (TRGP constructs, including active goals and important referents), separation knowledge and barriers. The latter topics are not addressed in this paper because they are included in a separate overall analysis of waste management; furthermore, it is worth mentioning that in the TPB and TRGP framework the effects of barriers are routed through PBC. All questions are based on a 7-point Likert scale or multiple-choice answers; most of the items utilize already validated scales; further information on constructs and measures are available in the supplementary information (SI).

All participants have been informed about the purpose of the study and the research has been conducted in an ethically correct manner in accordance with local statutory requirements.

### Data Collection and Analysis

Different approaches and software are available to calculate the minimum sample size (e.g., Slovin’s formula (SI), “G*Power” software which offers different types of statistical tests). A statistical power analysis performed with G*Power 3.1 (Faul et al. 2009) indicates a minimum sample size of 191 respondents (with significance criterion 0.05, power 0.80 (Cohen 1992), effect size 0.20).

A total of 223 respondents participated in the on-line questionnaire and 208 questionnaires were adequately filled out for the subsequent analysis (please refer to Table 1 for the list of indicators). The respondents were recruited in public places (e.g., shopping areas, railway stations, city streets) by random sampling. Data are analyzed with IBM Statistical Package for Social Sciences (SPSS) 26 and IBM AMOS 28 in order to perform descriptive statistics and SEM (Khan et al. 2021; Morrison et al. 2017). The 2-step analysis aims at first testing the model validity and reliability, followed by the assessment of the measurement and structural models to verify the predictive capabilities of TRGP in relation to separation behavior (Ling et al. 2018; Mondejar-Jimenez et al. 2016).

**Table 1:** Constructs and related Indicators

Constructs	Indicator Code	Indicators	Loadings	Cronbach’s α	Indicator Reliability	Average Variance Extracted
Active Procurement Goal (APG)	APG1	A clean(er) world is important to me	0.801	0.669	0.641	0.539
	APG2	I can contribute to a cleaner world by separating waste accurately on a daily basis	0.661		0.437	
Active approval Goal (AAG)	AAG3	To me, it is important if people around me approve my waste separation	0.520	0.624	0.270	0.529
	AAG4	I am supported in separating waste accurately on a daily basis by my important referent	0.888		0.789	
Attitude (ATT)	ATT1	My waste separation on a daily basis for the next 3 months is good/bad	0.748	0.744	0.560	0.506
	ATT2	My waste separation on a daily basis for the next 3 months is pleasant/unpleasant	0.637		0.406	
	ATT3	My waste separation on a daily basis for the next 3 months is useful/useless	0.744		0.554	
Subjective Norms (NOR)	NOR1	The most important person/group of people to me separates waste accurately on a daily basis	0.539	0.662	0.291	0.567
	NOR2	The most important person/group of people to me think that I should accurately separate waste on a daily basis	0.918		0.843	
Motivation (MOT)	MOT1	I am motivated to separate my waste accurately	0.897	0.816	0.805	0.621
	MOT2	Do you desire to separate waste accurately?	0.770		0.593	
Perceived Behavioral Control (PBC)	PBC1	If I wanted to, I am confident that I can accurately separate waste on a daily basis	0.738	0.762	0.545	0.621
	PBC2	It is my own conscious decision to accurately separate my waste on a daily basis	0.835		0.697	
Intention (INT)	INT1	I expect to separate my waste accurately on a daily basis	0.806	0.898	0.650	0.673
	INT2	I will separate my waste accurately on a daily basis	0.792		0.627	
	INT3	I intend to separate my waste accurately on a daily basis	0.861		0.741	

## Results

### Descriptive Statistics

The demographic sample consisted of 63.9% respondents (*n* = 133) from Zwolle and 36.1% (*n* = 75) from Maastricht. The sample shows a slight predominance of the age range 25 to 34 (27.4%), followed by a quite uniform distribution of the age ranges 35 to 44 (16.8%), 18 to 24 (15.4%), 45 to 54 (15.4%), 55 to 64 (13.9%); the sample at or above 65-years old is poorly represented. Other socio-demographic parameters have been investigated and results are shown in SI.

In relation to the education level of the sample, in both towns the largest portion holds a university degree, whereas a smaller portion attended high school or holds an associate degree, and a very limited number of respondents (*n* = 3) has an elementary education only. Therefore, the education level of the sample is quite high. The occupation status is quite different between the two towns: whereas in Maastricht there is a clear predominance of students compared to employees, in Zwolle the employees are significantly predominant.

Overall, the analysis of TRGP variables shows a reasonably normal distribution. The following analysis of data is based on the exclusion of cases pairwise and some extreme outliers.

### Statistical Analysis

A CFA based on the maximum likelihood estimation is performed with the IBM AMOS 28 software to assess the measurement model fit before proceeding to hypothesis testing. The maximum likelihood estimation (covariance-based SEM) is preferred to partial least square SEM considering this research is not exploratory but rather, it focuses on understanding the relationships among constructs (Khan et al. 2020; Wetzels et al. 2009).

The goodness of the measures is assessed in terms of indicator loadings, reliability and validity; in turn, the analysis of validity is made of construct validity and convergent validity. In this study indicator loadings are generally above the normal cutoff point of 0.700, although some indicators (APG2, AAG3, ATT2, NOR1) are in the range 0.500 and 0.700 (Table 1). In this case they are acceptable considering average variance extracted (AVE) is above 0.500 (Khan et al. 2020), although they require careful scrutiny. The scale score reliability measures the internal consistency through the widespread Cronbach’s alpha (α), which represents the “expected correlation between an actual test and a hypothetical alternative form of the same length” (Carmines and Zeller 1979, p. 45). The analysis of Cronbach’s α coefficient (Cronbach 1951) shows that all values are acceptable being above 0.500, although several authors like Nunnally and Bernstein (1978) suggest above 0.700. It is worth recalling Pallant (2020) who warns researchers that “Cronbach alpha values are, however, quite sensitive to the number of items in the scale” (p. 135), therefore we may expect values as low as 0.5 in scales made of very few items. In the case of a limited number of items in the scale, Pallant (2020) suggests checking “the mean inter-item correlation for the items” (p. 135) for optimal values between 0.2 and 0.4 (Briggs and Cheek 1986). Different approaches are also available for investigating scale reliability; Morrison et al. (2017) integrate Cronbach’s α analysis with the study of the indicator reliability (IR) or the composite reliability (CR), which is based on IR. IR is the “proportion of variance in each measured variable that is accounted for by the latent factor it supposedly represents” (Morrison et al. 2017, p. 1334); ideal values are above 0.39 (O’Rourke and Hatcher 2013). Therefore, we conclude that the internal consistency of all items is within acceptable limits although some indicators (AAG3, NOR1) are borderline (Taber 2018) (Table 1).

Convergent validity is measured by AVE; its cut-off value is 0.500 (Fornell and Larcker 1981); therefore, all latent variables meet this requirement.

The discriminant validity verifies that the constructs are different from each other. We apply the Fornell-Larcker criterion (Fornell and Larcker 1981), which states that discriminant validity is adequate when the square root of AVE (√AVE) per each construct is greater than the correlations with the other related constructs (Lin and Guan 2021). Table 5 of SI confirms that this criterion is verified for this case study.

Although some items present borderline values, we conclude that the measurement model is acceptable.

The model shows a root mean square error of approximation (RMSEA) of 0.116 (RMSEA LO 90% = 0.103, HI 90% = 0.128), which is normally out of tolerance, however it can be considered as a sufficient value for small size of the sample N. In fact, Chen et al. (2008)

“demonstrate that there is no empirical support for the use of 0.05 or 0.10 as universal cutoff values to determine adequate model fit. The means of the sampling distributions of the RMSEA are related to the size of the sample, the type of the model, and the degree of misspecification” (p. 476).

Taasoobshirazi and Wang (2016) also recommend researchers to be cautious with RMSEA values when dealing with limited samples; furthermore, “TLI [Tucker-Lewis index] and RMSEA indices reward for model parsimony and penalize for model complexity” (Perugini and Bagozzi 2001, p. 87). The CHI SQUARE test is 3.787; we usually aim for a value of 3.0 or below (Kline 2011), however Schumacker and Lomax (2004) accept values as high as 5.0, therefore we consider the model sufficiently fit considering the limited sample.

The analysis of hypotheses is presented in Table 2.

**Table 2:** Structural Equation Modeling (SEM) – Hypotheses testing

Hypothesis	Predictor		Dependent variable	*p*-value	*t-value*	*ß* (beta)	95% CI	Hypothesis Acceptance
H1a	APG (Active Procurement Goal)	––>	ATT	***	7.413	0.798	[0.617, 0.943]	Accepted
H1b	APG (Active Procurement Goal)	––>	MOT	0.002	3.212	0.494	[0.072, 0.953]	Accepted
H2a	AAG (Active Approval Goal)	––>	NOR	***	3.337	0.772	[0.642, 0.879]	Accepted
H2b	AAG (Active Approval Goal)	––>	MOT	0.297	1.049	0.170	[0.065, 0.370]	Rejected
H3	ATT (Attitude)	––>	MOT	0.003	3.070	0.445	[0.132, 0.851]	Accepted
H4	NOR (Subjective Norms)	––>	MOT	0.982	−0.024	−0.003	[−0.195, 0.225]	Rejected
H5	MOT (Motivation)	––>	INT	***	10.855	0.823	[0.640, 0.927]	Accepted
H6	PBC (Perceived Behavioral Control)	––>	INT	***	7.448	0.488	[0.201, 0.729]	Accepted

***p-value < 0.001. Confidence Intervals (CI) are based on 2000 sample bootstrapping procedure at 95% significance level

Table 2 clearly indicates that goals positively influence attitude and subjective norms at a statistically significant level; specifically, APG positively influences attitude to separate, *ß* = 0.798, *p* < 0.001, 95% CI = [0.617, 0.943], whereas AAG positively influences subjective norms *ß* = 0.772, *p* < 0.001, 95% CI = [0.642, 0.879]. Therefore, the hypotheses H1a and H2a are accepted. The situation is different in relation to the impact of goals on motivation; in fact, APG positively influences motivation at a statistically significant level (*ß* = 0.494, *p* = 0.002, 95% CI = [0.072, 0.953]), whereas AAG does not influence motivation at a statistically significant level (*ß* = 0.170, *p* = 0.297, 95% CI = [0.065, 0.370]). Therefore, hypothesis H1b is accepted and H2b is rejected.

The antecedents of motivation show different types of influence: attitude has a positive influence on motivation at a statistically significant level (*ß* = 0.445, *p* = 0.003, 95% CI = [0.132, 0.851]), whereas subjective norms have no statistically significant effect on motivation (*ß* = −0.003, *p* = 0.982, 95% CI = [−0.195, 0.225]). Therefore, hypothesis H3 is accepted and hypothesis H4 is rejected.

The antecedents of intentions influence the precursor of behavior at a statistically significant level; particularly, motivation which has a strong positive effect on intention (*ß* = 0.823, *p* < 0.001, 95% CI = [0.640, 0.927]). PBC shows an appreciable and positive effect on intention as well (*ß* = 0.488, *p* < 0.001, 95% CI = [0.201, 0.729]). Therefore, hypotheses H5 and H6 are accepted.

**Fig. 2:**
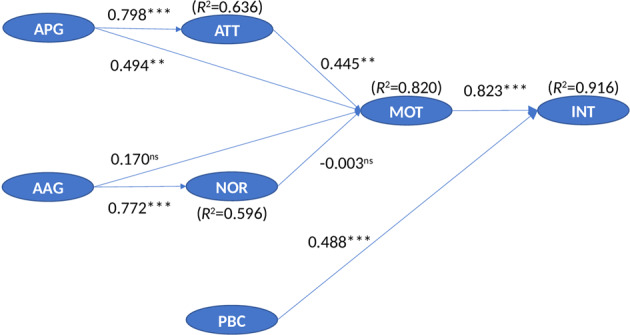
Theory of Reasoned Goal Pursuit (TRGP) Model – Structural Equation Modeling (SEM) estimation results (ß and R^2^). Note. **p *<* 0.005, ***p *<* 0.001, ns non-significant; all path coefficients are standardized

Furthermore, the model explains high levels of variance, namely 63.6% of variance in attitude (*R*^2^ = 0.636), 59.6% in subjective norms (*R*^2^ = 0.596), especially 82.0% in motivation (*R*^2^ = 0.820) and 91.6% in intention (*R*^2^ = 0.916) (Fig. 2). It is worth noticing that not only variance in intention is very high but also motivation (differently to Hamilton et al. (2022)’s case study). Although the definition of low/high (and acceptable/unacceptable) variance is quite debated (Brown et al. 1997; Nunnally and Berstein 1994; Peterson 2000; Tinsley and Tinsley 1987), the high variance in motivation may be related to the specificity of the sample. In terms of effect size (measured with Cohen’s D method), values indicate that the strength of the relationship between APG and motivation is strong (0.67), between APG and motivation is moderate (0.17), whereas the intensity of the relationships between AAG and motivation, or subjective norms and motivation are weak (respectively, 0.07 and 0.06).

## Discussion

Building on Ajzen and Kruglanski (2019, p. 777)’s consideration that “because most behaviors are goal-driven, their initiation presupposes the prior activation of one or more goals for which the behaviors in question serve as a means”, some researchers might object that recycling behavior is not really goal-driven. We admit that there are other volitional activities requiring strong concentration and determination in order to succeed; furthermore, nowadays recycling is a mandatory activity in many advanced economies. Therefore, one might think that no significant goal (or no goal at all) drives separation behavior. On the other hand, people living in advanced economies have developed an enhanced environmental awareness and sensitivity. Moreover, numerous studies demonstrate that repetitive behaviors may arouse some volitional mechanisms under specific circumstances during their execution. Ajzen and Kruglanski (2019, p. 781) infer that “routine behavior of this kind is not necessarily unintentional, although it may occur spontaneously, without a conscious intention”. Consequently, in the above-mentioned specific context, separating waste is goal-driven as well, therefore we expect a strong positive correlation among specific personal goals and other precursors of behaviors. Also, in specific conditions, motivation does arouse intention to perform a specific behavior; in fact, having the intention to separate does not automatically imply that the individual is going to perform waste separation. Therefore, we expect that the goal construct ultimately influences intention through its immediate precursor, namely motivation, which, in turn, is influenced by the typical TPB constructs of attitude and subjective norms. In this regard, it is also worth noticing that previous studies on recycling behavior in many advanced economy cities (Knussen et al. 2004; Mondejar-Jimenez et al. 2016) evidence the higher influence of attitude on intention compared to subjective norms, hence we anticipate a similar outcome in our case as well.

This paper actually shows that APGs have a significant direct effect on attitude and motivation to recycle, and an indirect effect on intention; AAGs strongly influence norms but have a statistically insignificant effect on motivation. These results may seem contradictory, but they are actually in line with our expectations for the above-mentioned generic reasons and for more specific arguments, explained below.

First, the construct of APG is somehow interconnected with attitude and, similarly, for AAG with subjective norms. In fact, Ajzen and Kruglanski (2019, p. 779) state that “activation of one or more procurement goals leads to consideration of behavioral options capable of attaining those goals. It follows that attitudes toward one or more behavioral options become relevant only in the context of active goals”. Moreover, the outcomes deriving from both AAG and APG have a predominant effect (“privileged status”) respectively, in the genesis of attitudes and subjective norms. Therefore, a strong behavioral or normative belief can significantly polarize attitude or subjective norms, regardless of the total effect of other existing beliefs which are dormant or not salient at that time. This aspect represents a significant change from TPB and

“stands in partial contrast to the compensatory nature of the expectance-value model of attitude in which behavioral beliefs of varying strength and valence can compensate for each other, and each product of belief strength times outcome evaluation is given equal weight” (Ajzen and Kruglanski 2019, p. 799).

Another key factor to consider is the overall stability of the environmental protection goals related to recycling and, specifically, to the separation activity at the household level, which normally takes place in quite standard conditions (e.g., visual cues, smell, position of bins).

Moreover, although highly volitional behaviors are, presumably, significantly goal-driven, less volitional or habitual behaviors are still driven by some goals; actually, Ajzen and Kruglanski (2019, p. 781) clearly state that “habitual behavior is typically goal-driven”. These goals may be less vivid because they are less persistent or latent, but they may be invigorated by contingent and contextual occurrences, like the action of removing food from a plastic container before throwing it in the correct bins. This simple and repetitive action may make you quickly think of the possibility of reducing plastic or paper waste by utilizing reusable packaging. These ordinary examples support Ajzen and Kruglanski (2019)’s assertion that “a strong habit may support some active goals” (p. 781). In addition, the results of our questionnaire show that the majority of participants consider environmental protection as a medium to high importance goal.

As expected, our results confirm that, in the case of separation behavior, AAG does not reach the level of importance as APG. If we take a closer look at the social context, we realize that a correct and diligent waste separation behavior does not significantly contribute to the individual’s social recognition, especially in the case of a common conviction of poor recycling services or lack of sanctioning for improper separation. Therefore, the limited approval by the social group of reference is sufficient not to promote approval goals, which in turn do not directly contribute to motivation (*ß* = 0.170, *p* = 0.297, 95% CI = [0.065, 0.370]), nor indirectly through subjective norms.

The analysis of the effect of motivation on intention confirms that motivation is the immediate precursor of intention. This new construct represents a step forward for TPB; in fact, TRGP introduces a construct that measures the desirability and attainability of a goal. In this regard, Ajzen and Kruglanski (2019) highlight that “action is unlikely to be initiated unless the goal is sufficiently desirable and its perceived likelihood of attainment exceeds a certain threshold level” (p. 777). In fact, they explain that, although an individual may have a positive attitude toward recycling, and he/she feels the social pressure to recycle, the individual does not automatically form the intention to recycle nor perform recycling. To do that, the individual definitely needs to understand that separating waste is a means to achieve one or more active goals such as environmental ones. It is also worth mentioning that environmental motivation does not fluctuate over time and it is directly related to recycling behavior (Otto et al., 2018); these aspects clearly favor the application of TRGP on recycling behavior.

In line with our expectations and with the TPB, PBC represents a key construct when analyzing recycling intention and behavior. Our paper confirms the key role of PBC on intention as well (*ß* = 0.488, *p* < 0.001, 95% CI = [0.201, 0.729]), and it also indicates that PBC is not influenced by goals because this construct “refers to people’s expectancy that their attempts to execute the behavior will be successful” (Ajzen and Kruglanski 2019, p. 780). At the same time, the perceived individual ability expressed through PBC enables the possibility of attaining one or more (procurement or approval) goals.

Looking at the precursors of motivation, the effect of norms on motivation is not comparable to the effect of attitude on the same construct. This outcome is in accordance with our expectations because in this recycling context the effects of social norms are quite limited, whereas the attitude towards recycling represents a key factor.

In any case, this TRGP model displays high levels of *R*^2^ for attitude (63.6%), norms (59.6%) and, especially, motivation (82.0%) and intention (91.6%), indicating the significant predictive validity of this framework when applied to separation behavior.

## Conclusions

This paper investigates the effectiveness of the TRGP in explaining waste separation behavior at the household level in two medium-size cities in the Netherlands, where recycling represents a well-established procedure. Moreover, this paper represents a seminal application of TRGP in the field of environmental behaviors, thus contributing to a new line of research on recycling behavior.

The results of this study indicate that integrating the goal construct within TPB improves the explanatory power of TPB and supports the validity of TRGP as a framework for analyzing recycling behavior. We concur with Ajzen and Kruglanski (2019, p. 777) that this addition makes “explicit what hitherto was only implicit in TPB-guided behavioral explorations”. TRGP moves a step ahead of TPB by acknowledging that “behaviors are usually performed in the service of certain goals” (Ajzen and Kruglanski 2019, p. 777). Considering waste separation at the household level usually takes place in a stable context and in a repetitive manner, at a first approach we may expect a limited influence of goals on the intention to separate. Actually, the effects of APG are statistically significant both on recycling attitude and motivation. Consequently, this paper highlights that TRGP exhibits a strong explanatory power for behavior not under full volitional control as well, as in the case of habitual behaviors. Moreover, the results indicate that motivation represents a very reliable proxy of intention to separate; in fact, the construct of motivation is able to improve the predictive capabilities of TPB by explaining why a strong intention to recycle does not automatically form the recycling behavior unless it is supported by adequate motivation. Specifically, having the intention to separate does not automatically imply that the individual is going to perform waste separation. At the same time, if an individual has a positive attitude toward recycling and he/she feels the social pressure to perform recycling, the individual does not automatically form the intention to recycle unless he/she has the motivation to achieve an active goal. Therefore, as expected, our findings support the addition of active goals and motivation as precursors of intention; in particular, in the case of waste separation, the construct of APG enhances the predictability of separation intention.

### Implications and Policy Suggestions

The outcomes of this case study provide useful indications to interventionists about effective measures to promote behavior changes to improve waste separation. The key role of goals and motivation calls for more targeted interventions. It becomes essential to activate the applicable goals and means; in fact, empirical studies indicate that “when a goal is activated, competing goals are inhibited” (Kruglanski and Szumowska 2020, p. 1266), and similarly for means. Waste service providers, municipalities and higher institutions should aim at developing persuasive communication (Hamilton et al. 2022) and promoting high level goals. As explained by Kruglanski and Szumowska (2020), “treating habits as instances of goal-directed behavior also has important implications for the possibility of changing habits and uprooting ones that are undesirable or harmful” (p. 1266). This statement particularly applies to recycling behavior which is often characterized by improper separation routines. In this case, interventionists should first identify the goals serving the correct behavior (Kruglanski et al. 2002), define the alternative behavior and pair it with the desired goals in order to create an expectancy that the alternative behavior serves the goals in a more effective way (Kruglanski and Szumowska 2020).

Although this paper highlights the importance of APG, interventionists should promote AAG as well. In this case, the spectrum of intervention is quite wide because it ranges from the family dimension to the society level, including schools and workplace. Promotion of approval goals should be pursued in combination with the enhancement of subjective norms by encouraging the approval of important others or by fostering social recognition (Hamilton et al. 2022). We expect these interventions have to be tailored depending on socio-demographic characteristics such as age, because elders are likely to have a different goals system from youngsters (SI).

### Future Research Directions

This paper offers some suggestions for future research directions.

Considering TRGP extends the range of applications of TPB (Ajzen and Kruglanski 2019), it would be useful to test the predictive capability of TRGP in different contexts. As inferred by Fishbein and Ajzen (2011) when defending the TPB solidity, a solid theoretical framework has to undergo the test of generalization.

Furthermore, TRGP may not represent a theoretical end state, thus scholars are invited to further explore this theoretical framework with possible additions or modifications. In fact, (Ajzen 2015) states that “there is nothing in the TPB to preclude addition of new predictors. Indeed, the TPB was developed by adding perceived behavioural control to the original theory of reasoned action” (p. 2). In any case, these modifications need to be well justified, including the sufficiency assumptions (Fishbein and Ajzen 2011).

A comparative analysis between similar samples of population from different cities may offer the possibility of better understanding recycling behavior and the effectiveness of recycling procedures. Furthermore, a multi-group analysis of the sample based on socio-demographic characteristics (e.g., age, income) may help in defining more targeted interventions.

Moreover, this paper highlights the importance of conducting a correct analysis of active goals, in particular when dealing with habitual behaviors. In fact, habits may conceal the real presence of goals and lead to the wrong conclusion of a lack of pertinent active goals. Researchers should also carefully consider the intrinsic limitations of questionnaires when analyzing active goals not adequately elicited by the method of investigation.

### Limitations

We also acknowledge some limitations in our study.

Measures are not taken from actual observations, but they are based on self-reported information; furthermore, this research does not measure behavior itself, but it actually analyzes the precursors of behavior. Therefore, data based on real observation may provide different outcomes from data coming from reported behavior (Ali and Ahmad 2016); moreover, this paper does not contribute to the literature on intention-behavior gap. The limited sample exposes collected data and related scales to the risk of internal inconsistency, high RMSEA, low CFI (Comparative Fit Index) and similar indexes. In addition, our sample may be biased by the COVID-19 pandemic and the slight predominance of the age range 25–34. Therefore, this sample does not entirely represent the population of the two cities in relation to the age of respondents. Lastly, although Otto et al. (2018) report that environmental motivation is a relatively stable actor, there is the real risk that the fluctuations of goals and motivation are not adequately captured during the data collection phase.

### Supplementary Information

Supplementary Information (SI)
