# Supplementary material for: Investigating the Role of Goals and Motivation on Waste Separation Behavior Through the Lens of the Theory of Reasoned Goal Pursuit
Source: Environ Manage. 2023 May 3;72(5):1019–31. doi: 10.1007/s00267-023-01820-1 (PMC10154762; doi:10.1007/s00267-023-01820-1)
Supplement: Supplementary file 1 — Supplementary Information (SI) [file 267_2023_1820_MOESM1_ESM.docx]

Supplementary Information (SI)

Literature Review and Theoretical Framework

Several meta-analysis (Miafodzyeva & Brandt, 2013; Xianfang et al., 2017), systematic literature reviews (Concari et al., 2020) and knowledge domain mappings (Concari et al., 2022) have investigated the main socio-psychological frameworks applied to recycling behavior in the last decades. They clearly show a predominance of a cognitive reasoned approach framework, which mainly explains pro-environmental intention and behavior through constructs like individual attitude, influence of norms, perception of being able to control the situation, beliefs, evaluation of consequences, awareness of the situation and ascription of responsibility. In particular, numerous scholars have focused on the discrepancy between behavior and its precursors, namely the “attitude-behavior gap” (Carmi et al., 2015), the “intention-behavior gap” (Carrington et al., 2010, 2014; Zhang & Zhao, 2019), the “intention action gap” (de Koning et al., 2016), or the “attitude-behaviour discrepancy” (Eiser & Eiser, 1986). Other constructs as emotions, desire, motivation and goals have been often kept out of the recycling behavior equation (Carrus et al., 2008; Damasio, 1998; Geng et al., 2017; Perugini & Bagozzi, 2001).

Model of Goal-directed Behavior (MGB)

In MGB the immediate predictor of intention is desire which “mediates the effects of attitude, subjective norms, PBC and anticipated emotions on intention and behavior” (Parkinson et al., 2018, p. 840); at the same time, PBC does not directly influence intention but desire and behavior. Considering some scholars criticize TPB for not explaining “how intentions become energized” (Perugini & Bagozzi, 2001, p. 83), Perugini and Bagozzi (2001) introduce desire as “the motivational impetus for intention” (p. 83); in turns, attitude, subjective norms and PBC are the catalyst to fire up the dormient desire. Another important additional construct is anticipated emotions which are the referents of personal goals; in fact, Perugini and Bagozzi (2001, p. 83) state that “anticipated emotions function as independent variables based upon a decision process that takes into account judged consequences of goal achievement and goal failure”. Furthermore, Perugini and Bagozzi (2001), consider (frequency of) past behavior as a predictor of desire, intention and behavior; on the contrary, Ajzen infers that the residual effects of past behavior are mediated by PBC. In relation to the application of MGB to recycling behavior Carrus et al. (2008) find out a consistent relationship between negative anticipated emotions and desire to recycle; moreover, this relationship is more statistically significant than the one between attitude and desire, or PBC and desire.

Norm Activation Model (NAM)

In NAM, in order to perform a pro-social behavior, the individual has to be conscious that an anti-social behavior leads to negative consequences towards others; furthermore, the individual needs to understand its consequent responsibilities towards the society or its group of reference. Overall, NAM focuses on the key role of personal norms, which can be activated in different forms. Some scholars as De Groot and Steg (2009) proved the validity of this framework in predicting pro-social intention and behavior in different environmental contexts.

Other Theoretical Frameworks

Other theories and models have been applied to recycling behavior, either in combination with the mentioned frameworks or alone. For example, the Neutralization Theory of Delinquency (Sykes & Matza, 1957) has been applied by Tang et al. (2011) through the construct of justification in combination with TPB. Hansmann et al. (2006) have also created a model including justification, socio-demographic variables, knowledge, attitude, social norms and recycling behavior. In this case Hansmann et al. (2006) combine justification with constructs coming from different models, such as TPB and NAM. They interpret justification in terms of ascribed responsibility and awareness of consequences; they also infer that adding “justification for non-recycling” to the analysis of this type of behavior enhances “the explanatory power of models predicting recycling behavior” (p. 156). Other scholars like Qin and Song (2022) and Zhang et al. (2021) combine TPB with the Attitude-Behavior-Context (ABC) model by Guagnano et al. (1995). Gan and Zhang (2020) add the Interpersonal Behavior Theory (Triandis, 1977) to TPB as well.

It is also worth noticing that, besides the above-mentioned cases, TPB and NAM have been combined in the same framework with positive outcomes. For example, Park and Ha (2014) confirmed the influence of personal norms, attitude and PBC on the intention to recycle. Wang et al. (2018) analyze the influence of information publicity on intention to e-waste recycling and they conclude that information publicity indirectly influence intention through attitude and personal norms.

Goal Setting Theory

The Goal Setting Theory concentrates on the individual who sets its own goal to fulfill its needs. These needs are clearly influenced by further factors like attitude towards the goals; similarly, personal goals are affected by social beliefs. Latham et al. (2011) consider goal as “an object or aim that an individual strives to attain”; moreover, “goals are the immediate regulators of behavior”. Considering this theory has been created to analyze the individual motivation at work, it defines four mediators which positively influence the goal: choice, effort, persistence and strategy. Given the specific focus on workplace (Ciocirlan, 2017; Ciocirlan et al., 2020; Kollmuss & Agyeman, 2002; Norton et al., 2015), the academic literature does not show examples of application at the household or consumer level. In any case, “findings revealed that the corporate environmental strategy significantly explains the psychological green climate, which, in turn, enhances voluntary environmental behavior, like energy-saving behavior, waste reduction behavior, and resource recycling behavior” (Das et al., 2019).

Goal Systems Theory (GST)

As explained in the main paper, Kruglanski et al. (2015) utilize the concepts of multifinality, equifinality and counterfinality. In fact, a goal can be reached by one or more means, and, vice versa, one single mean can satisfy one or more goals; furthermore, a goal can represent a top priority in our life or can compete with other objectives at other times.

Another important aspect of the GST is that goal systems have motivational and cognitive properties (Kruglanski et al., 2002). The former are driven by the “principle of subjective utility, which determines goal-commitment and mean choice” (Kruglanski et al., 2002, p. 342); moreover, the strive for a goal is influenced by persistence of pursuit and affective feedback. The latter are characterized by structural and allocational properties, namely the type of links between goals and means (interconnectedness), and the mental resources availability in a “constant sum” game. Being said that the cognitive properties often take over the motivational ones, goals may range from short terms and narrow objective to long term ambitions (Kruglanski et al., 2002).

Methodology

Research Design – Sample Definition, Data Collection and Cleaning

The Slovin’s formula (De Feo et al., 2017; Dhokhikah et al., 2015) has also been considered to determine the sample size (n=sample number, N=total population, e=margin of error)

n = $\frac{N}{Ne^{2}+1}$

In year 2018 households in Maastricht and Zwolle are respectively 69,180 and 58,546, therefore the corresponding samples are respectively 69.37 and 69.36 respondents (with a margin of error of .12).

Please note that the study of the relationship between the socio-demographic variables (e.g., age, gender) and socio-psychological constructs is out of the scope of this paper.

As explained in the main paper some questionnaires were dropped because of missing data and outliers; outliers “can have a dramatic effect on the correlation coefficient, particularly in small samples” (Pallant, 2020, p. 159). In this regard, it is worth recalling Osborne (2010): “an extreme score, or data point far outside the normal distribution for a variable or population, is also described as an observation that deviates so much from other observations as to arouse suspicious that it was generated by a different mechanism” (p. 37). Although in the academic literature there is a lot of controversy over what represents an outlier, several scholars apply the rule of thumb of scores of 3 or more SDs (from the mean) when deciding whether further examining critical data (Osborne, 2013). Osborne (2010) suspects that “data points beyond +/- 3 SD from the mean are not generated from the population of interest” (p. 37). In our study we applied both statistical and graphical methods (through IBM SPSS) for investigating extreme scores; for example, we analyzed indices such as Mahalanobis’s distance (Osborne & Overbay, 2004) and scatterplots such as the normal P-P plot of regression standardized residual (Pallant, 2020). Considering that there may be different causes for extreme scores and the “inferred cause can then inform what action a researcher should take” (Osborne, 2010, p. 38), our analysis supports the possibility of extreme scores in our sample due to intentional misreporting and “legitimate cases sampled from the correct population” (Osborne, 2010, p. 39). In particular, intentional misreporting by respondents could be motivated by social desirability or self-presentation (Osborne, 2010); for example, high levels of motivation or intention to separate waste versus a low frequency of waste separation for all types of waste. Extreme legitimate cases are represented by respondents with strong recycling attitude and intention but not recycling some type of waste (e.g., glass bottle); this may happen when respondents regularly separate their waste and purchase food or drink contained in specific types of container (e.g., plastic bottle or paper box). In the case of misreporting, we followed Osborne (2010)’s recommendation to remove those respondents.

Socio-psychological Constructs and Related Measures

The socio-psychological constructs are described as follows:

- *Active Procurement Goals* (AGPs) are measured through two indicators asking respondents if a cleaner world is important to them (APG1), and if they can contribute to a cleaner world by separating waste accurately on a daily basis (APG2), on 7-point Likert scale ranging from “True (1)” to “False (7)” (with “Neutral (4)”).
- *Active Approval Goals* (AGPs) are measured through two indicators asking respondents if it is important that people (around them) approve their waste separation (AAG3), and if they are supported in separating waste accurately on a daily basis by applicable important others (AAG4), on 7-point Likert scale ranging from “True (1)” to “False (7)” (with “Neutral (4)”). Before posing the previous question, and in order to define the important referents, a specific multiple-choice question asks respondents to the define the important others (“Who’s approval is important to you”). The possible choices are based on the eliciting questionnaire: partner, closest friend(s), family, neighbor (s), government, others to specify.
- *Attitude* (ATT) is measured by three items asking whether the respondent daily waste separation (for the next 3 months) is bad or good (ATT1), pleasant/unpleasant (ATT2), useful/useless (ATT3), on a 7-point Likert scale ranging from fully positive to fully negative.
- *Subjective norms* (NOR) are measured by two items asking: “The most important person/group of people to me separates waste accurately on a daily basis” (NOR1) and “The most important person/group of people to me think that I should accurately separate waste on a daily basis” (NOR2) on 7-point scale ranging from fully agree to fully disagree.
- *Motivation* (MOT) is measured by two items asking: “I am motivated to separate my waste accurately” (MOT1) and “Do you desire to separate waste accurately?” (MOT2), on a 7-point Likert scale ranging from “True (1)” to “False (7)” (with “Neutral (4)”).
- *Perceived Behavioral Control* (PBC) is measured by two items asking: “If I wanted to, I am confident that I can accurately separate waste on a daily basis” (PBC1) and “It is my own conscious decision to accurately separate my waste on a daily basis” (PBC2), on a 7-point Likert scale ranging from “True (1)” to “False (7)” (with “Neutral (4)”).
- *Intention* (INT) was measured through three items asking participants whether they expect/will/intend (INT1, INT2, INT3) to separate waste on a daily basis, on a 7-point Likert scale ranging from “True (1)” to “False (7)” (with “Neutral (4)”).

It is worth noticing the questionnaire asked three or four questions (items) per each construct, but some items have been dropped out because of a reduced loading.

Table 1 indicates the source as well (as applicable).

Table 1 – Constructs and Sources

| Constructs | Indicator Code | Indicators | Source |
| --- | --- | --- | --- |
| Active Procurement Goal (APG) | APG1 | A clean(er) world is important to me | // |
|  | APG2 | I can contribute to a cleaner world by separating waste accurately on a daily basis | // |
| Active Approval Goal (AAG) | AAG3 | To me, it is important if people around me approve of my waste separation | // |
|  | AAG4 | I am supported in separating waste accurately on a daily basis by my important referent | // |
| Attitude (ATT) | ATT1 | My waste separation on a daily basis for the next three months is good/bad | (Ajzen, 2006) |
|  | ATT2 | My waste separation on a daily basis for the next three months is pleasant/unpleasant | (Ajzen, 2006) |
|  | ATT3 | My waste separation on a daily basis for the next three months is useful/useless | (Ajzen, 2006) |
| Subjective Norms (NOR) | NOR1 | The most important person/group of people to me separates waste accurately on a daily basis | (Ajzen, 2006) |
|  | NOR2 | The most important person/group of people to me think that I should accurately separate waste on a daily basis | (Ajzen, 2006) |
| Motivation (MOT) | MOT1 | I am motivated to separate my waste accurately | (Gamba & Oskamp, 1994)  (Otto et al., 2018) |
|  | MOT2 | Do you desire to separate waste accurately? | (Gamba & Oskamp, 1994)  (Otto et al., 2018) |
| Perceived Behavioral Control (PBC) | PBC1 | If I wanted to, I am confident that I can accurately separate waste on a daily basis | (Ajzen, 2006) |
|  | PBC2 | It is my own conscious decision to accurately separate my waste on a daily basis | (Ajzen, 2006) |
| Intention (INT) | INT1 | I expect to separate my waste accurately on a daily basis | (Ajzen, 2006) |
|  | INT2 | I will separate my waste accurately on a daily basis | (Ajzen, 2006) |
|  | INT3 | I intend to separate my waste accurately on a daily basis | (Ajzen, 2006) |

Results

Descriptive Statistics

The below tables integrate the main manuscript as follows:

- Table 2 provides an overall statistic description of the overall sample
- Table 3 describes the samples per each town
- Table 4 presents *M* and *SD* of constructs
- Table 5 presents the correlation matrix

Table 2 – Overall Description of Sample (Zwolle and Maastricht respondents)

| Variables | Answers  (values for *M* and *SD* calculation) | *M* | *SD* | Frequency  n | Percentage  % |
| --- | --- | --- | --- | --- | --- |
| Respondents |  |  |  | 208 | 100% |
| Age Range |  | 3.2 | 1.6 |  |  |
|  | 18-24 (1) |  |  | 32 | 15.4% |
|  | 25-34 (2) |  |  | 57 | 27.4% |
|  | 35-44 (3) |  |  | 35 | 16.8% |
|  | 45-54 (4) |  |  | 32 | 15.4% |
|  | 55-64 (5) |  |  | 29 | 13.9% |
|  | 65-74 (6) |  |  | 19 | 9.1% |
|  | 75-84 (7) |  |  | 4 | 1.9% |
| Gender |  | 0.9 | 0.7 |  |  |
|  | Male (0) |  |  | 59 | 28.4% |
|  | Female (1) |  |  | 134 | 64.4% |
|  | Other (2) |  |  | 1 | 0.5% |
|  | Prefer not to answer/specify (3) |  |  | 14 | 6.8% |
| Education |  | 3.3 | 1.1 |  |  |
|  | Different cases/not specified (0) |  |  | 13 | 6.3% |
|  | Elementary/primary school (1) |  |  | 3 | 1.4% |
|  | High school/secondary school (2) |  |  | 21 | 10.1% |
|  | Associate degree (3) |  |  | 51 | 24.5% |
|  | University education (4) |  |  | 120 | 57.7% |
| Type of dwelling |  | 1.4 | 0.8 |  |  |
|  | Other (0) |  |  | 42 | 20.2% |
|  | Flat (1) |  |  | 45 | 21.6% |
|  | House (2) |  |  | 121 | 58.2% |
| Employment status |  | 6.5 | 2.6 |  |  |
|  | Other/Not specified (0) |  |  | 14 | 6.8% |
|  | Unemployed (not looking for a job) (1) |  |  | 5 | 2.4% |
|  | Unemployed (looking for a job), Student (2) |  |  | 2 | 1.0% |
|  | Unemployed (looking for a job) (3) |  |  | 8 | 3.8% |
|  | Disabled (4) |  |  | 6 | 2.9% |
|  | Student (5) |  |  | 29 | 13.9% |
|  | Employed part time, Student (6) |  |  | 5 | 2.4% |
|  | Employed part time (7) |  |  | 56 | 26.9% |
|  | Retired (8) |  |  | 20 | 9.6% |
|  | Employed full time (9) |  |  | 63 | 30.3% |

Table 3 - Description of Sample by town

| Variables | Answers | Zwolle  Frequency  n | Zwolle  Percentage % | Maastricht  Frequency n | Maastricht  Percentage % |
| --- | --- | --- | --- | --- | --- |
| Respondents |  | 133 | 63.9% | 75 | 36.1% |
| Age Range |  |  |  |  |  |
|  | 18-24 | 13 | 6.3% | 19 | 9.1% |
|  | 25-34 | 33 | 15.9% | 24 | 11.5% |
|  | 35-44 | 30 | 14.4% | 5 | 2.4% |
|  | 45-54 | 25 | 12.0% | 7 | 3.4% |
|  | 55-64 | 18 | 8.7% | 11 | 5.3% |
|  | 65-74 | 13 | 6.3% | 6 | 2.9% |
|  | 75-84 | 1 | 0.5% | 3 | 1.4% |
| Gender |  |  |  |  |  |
|  | Male | 28 | 37.3% | 31 | 23.3% |
|  | Female | 42 | 56.0% | 92 | 69.2% |
|  | Other | 0 | 0.0% | 1 | 0.8% |
|  | Prefer not to answer/ specify | 9 | 6.7% | 5 | 6.7% |
| Education |  |  |  |  |  |
|  | Different cases/not specified | 8 | 6.0% | 5 | 6.6% |
|  | Elementary/primary school | 2 | 1.5% | 1 | 1.3% |
|  | High school/secondary school | 10 | 7.5% | 11 | 14.7% |
|  | Associate degree | 39 | 29.3% | 12 | 16.0% |
|  | University education | 74 | 55.6% | 46 | 61.3% |
| Type of dwelling |  |  |  |  |  |
|  | Other | 33 | 24.9% | 9 | 12% |
|  | Flat | 26 | 19.5% | 19 | 25.3% |
|  | House | 74 | 55.6% | 47 | 62.7% |
| Employment status |  |  |  |  |  |
|  | Other | 8 | 5.9% | 6 | 7.9% |
|  | Unemployed  (not looking for a job) | 2 | 1.5% | 3 | 4.0% |
|  | Unemployed  (looking for a job), Student | 0 | 0.0% | 2 | 2.7% |
|  | Unemployed  (looking for a job) | 3 | 2.3% | 5 | 6.7% |
|  | Disabled | 5 | 3.8% | 1 | 1.3% |
|  | Student | 9 | 6.8% | 20 | 26.7% |
|  | Employed part time, Student | 2 | 1.5% | 3 | 4.0% |
|  | Employed part time | 44 | 33.1% | 12 | 16.0% |
|  | Retired | 14 | 10.5% | 6 | 8.0% |
|  | Employed full time | 46 | 34.6% | 17 | 22.7% |

Table 4 - Overall Description of Constructs

| Constructs | Indicator Code | Range | *M* | *SD* |
| --- | --- | --- | --- | --- |
| Active Procurement Goal | APG | 1-7 | 2.3 | 1.3 |
| Active Approval Goal | AAG | 1-7 | 4.1 | 1.7 |
| Attitude | ATT | 1-7 | 2.4 | 1.3 |
| Subjective Norms | NOR | 1-7 | 2.8 | 1.4 |
| Motivation | MOT | 1-7 | 1.9 | 1.2 |
| Perceived Behavioral Control | PBC | 1-7 | 2.0 | 1.3 |
| Intention | INT | 1-7 | 2.2 | 1.3 |

Table 5- Discriminant validity using Fornell-Larcker Criterion

|  | APG | MOT | INT | PBC | AAG | ATT | SN |
| --- | --- | --- | --- | --- | --- | --- | --- |
| APG | **.734** |  |  |  |  |  |  |
| MOT | .654 | **.816** |  |  |  |  |  |
| INT | .630 | .809 | **.820** |  |  |  |  |
| PBC | .578 | .640 | .750 | **.788** |  |  |  |
| AAG | .260 | .331 | .271 | .268 | **.727** |  |  |
| ATT | .581 | .674 | .678 | .526 | .280 | **.711** |  |
| SN | .358 | .426 | .478 | .393 | .482 | .375 | **.753** |

Note: The values (in bold characters) along the diagonal indicate the √AVE of the latent variable.

Discussion

Goals Fluctuation

The topic of goal fluctuation deserves adequate attention in the analysis of human behavior, especially considering that people are often guided by different goals and, at times, a specific goal takes over other goals for a multitude of reasons. The individual mood, high-level of stress, the influence of important others or media, contextual conditions, etc. may explain the selection of a goal in spite of another one. In the specific case of environmental goals, we acknowledge that, although goals are often fluctuating and conflicting, the global critical environmental situation contributes to maintain a continuous high level of attention on topics like energy-saving, resource consumption, pollution, waste and circular economy; these topics are daily addressed on the news, on magazines and on social media as well. Therefore, we expect that environmental goal has a limited fluctuation for people living in advanced economies.

Conclusions

Implications and Policy Suggestions

As explained in the main paper, governmental interventions need to be adequately tailored on the targeted audience, especially from the socio-demographical point of view. For example, in relation to age, the youngsters need appropriate school programs including environmental education since the early stages, whereas the elders require a different approach considering they may have inappropriate recycling habits, they me reluctant to implement new separation procedures or they may lack of environmental motivation. Numerous behavior change techniques are available depending on the situation (Abraham & Michie, 2008). In the former case the environmental education needs to be supported by prompting specific goal setting, demonstration of appropriate behavior, techniques of intention formation, encouragement and rewards. In the latter case behavior changes may be pursued by providing information on health risks and conducting motivational interviewing. Explaining benefits and costs for proper and improper action may prove beneficial as well.

References

Abraham, C., & Michie, S. (2008). A taxonomy of behavior change techniques used in interventions. *Health psychology, 27*(3), 379. <https://doi.org/10.1037/0278-6133.27.3.379>

Ajzen, I. (2006). *Constructing a TPB questionnaire: Conceptual and methodological considerations*. Retrieved 18.02.2023 from <https://citeseerx.ist.psu.edu/document?repid=rep1&type=pdf&doi=0574b20bd58130dd5a961f1a2db10fd1fcbae95d>

Carmi, N., Arnon, S., & Orion, N. (2015). Seeing the forest as well as the trees: General vs specific predictors of environmental behavior. *Environmental Education Research, 21*(6), 1011-1028. <https://doi.org/10.1080/13504622.2014.949626>

Carrington, M. J., Neville, B. A., & Whitwell, G. J. (2010). Why Ethical Consumers Don’t Walk Their Talk: Towards a Framework for Understanding the Gap Between the Ethical Purchase Intentions and Actual Buying Behaviour of Ethically Minded Consumers. *Journal of Business Ethics, 97*(1), 139-158. <https://doi.org/10.1007/s10551-010-0501-6>

Carrington, M. J., Neville, B. A., & Whitwell, G. J. (2014). Lost in translation: Exploring the ethical consumer intention–behavior gap. *Journal of Business Research, 67*(1), 2759-2767. <https://doi.org/10.1016/j.jbusres.2012.09.022>

Carrus, G., Passafaro, P., & Bonnes, M. (2008). Emotions, habits and rational choices in ecological behaviours: The case of recycling and use of public transportation. *Journal of Environmental Psychology, 28*(1), 51-62. <https://doi.org/10.1016/j.jenvp.2007.09.003>

Ciocirlan, C. E. (2017). Environmental Workplace Behaviors:Definition Matters. *Organization & Environment, 30*(1), 51-70. <https://doi.org/10.1177/1086026615628036>

Ciocirlan, C. E., Gregory‐Smith, D., Manika, D., & Wells, V. (2020). Using Values, Beliefs, and Norms to Predict Conserving Behaviors in Organizations. *European Management Review, 17*(2), 543-558. <https://doi.org/10.1111/emre.12388>

Concari, A., Kok, G., & Martens, P. (2020). A Systematic Literature Review of Concepts and Factors Related to Pro-Environmental Consumer Behaviour in Relation to Waste Management Through an Interdisciplinary Approach. *Sustainability, 12*(11), 4452. <https://doi.org/10.3390/su12114452>

Concari, A., Kok, G., & Martens, P. (2022). Recycling behaviour: Mapping knowledge domain through bibliometrics and text mining. *Journal of Environmental Management, 303*, 114160. <https://doi.org/10.1016/j.jenvman.2021.114160>

Damasio, A. R. (1998). Emotion in the perspective of an integrated nervous system. *Brain research reviews, 26*(2-3), 83-86. <https://doi.org/10.1016/S0165-0173(97)00064-7>

Das, A. K., Biswas, S. R., Abdul Kader Jilani, M. M., & Uddin, M. A. (2019). Corporate environmental strategy and voluntary environmental behavior—Mediating effect of psychological green climate. *Sustainability, 11*(11), 3123. <https://doi.org/10.3390/su11113123>

De Feo, G., Polito, A. R., Ferrara, C., & Zamballetti, I. (2017). Evaluating opinions, behaviours and motivations of the users of a MSW separate collection centre in the town of Baronissi, Southern Italy. *Waste Management, 68*, 742-751. <https://doi.org/10.1016/j.wasman.2017.06.045>

De Groot, J. I. M., & Steg, L. (2009). Morality and Prosocial Behavior: The Role of Awareness, Responsibility, and Norms in the Norm Activation Model. *The Journal of Social Psychology, 149*(4), 425-449. <https://doi.org/10.3200/SOCP.149.4.425-449>

de Koning, J. I. J. C., Ta, T. H., Crul, M. R. M., Wever, R., & Brezet, J. C. (2016). GetGreen Vietnam: towards more sustainable behaviour among the urban middle class. *Journal of Cleaner Production, 134*, 178-190. <https://doi.org/10.1016/j.jclepro.2016.01.063>

Dhokhikah, Y., Trihadiningrum, Y., & Sunaryo, S. (2015). Community participation in household solid waste reduction in Surabaya, Indonesia. *Resources Conservation and Recycling, 102*, 153-162. <https://doi.org/10.1016/j.resconrec.2015.06.013>

Eiser, J. R., & Eiser, J. R. (1986). *Social psychology: Attitudes, cognition and social behaviour*. Cambridge University Press.

Gamba, R. J., & Oskamp, S. (1994). Factors influencing community residents' participation in commingled curbside recycling programs. *Environment and Behavior, 26*(5), 587-612. <https://doi.org/10.1177/0013916594265001>

Gan, B., & Zhang, C. (2020). Influencing Factors of Urban Residents’ Garbage Classification and Recycling Behavior Driving Mechanism in Artificial Intelligence Environment. *IOP Conference Series: Earth and Environmental Science, 619*(1), 012006. <https://doi.org/10.1088/1755-1315/619/1/012006>

Geng, J., Long, R., Chen, H., & Li, W. (2017). Exploring the motivation-behavior gap in urban residents’ green travel behavior: A theoretical and empirical study. *Resources, Conservation & Recycling, 125*, 282-292. <https://doi.org/10.1016/j.resconrec.2017.06.025>

Guagnano, G. A., Stern, P. C., & Dietz, T. (1995). Influences of attitude-behavior relationships: A natural experiment with curbside recycling. *Environment and Behavior, 27*(5), 699-718. <https://doi.org/10.1177/0013916595275005>

Hansmann, R., Bernasconi, P., Smieszek, T., Loukopoulos, P., & Scholz, R. W. (2006). Justifications and self-organization as determinants of recycling behavior: The case of used batteries. *Resources, Conservation and Recycling, 47*(2), 133-159. <https://doi.org/10.1016/j.resconrec.2005.10.006>

Kollmuss, A., & Agyeman, J. (2002). Mind the Gap: Why do people act environmentally and what are the barriers to pro-environmental behavior? *Environmental Education Research, 8*(3), 239-260. <https://doi.org/10.1080/13504620220145401>

Kruglanski, A. W., Chernikova, M., Babush, M., Dugas, M., & Schumpe, B. M. (2015). Chapter Three - The Architecture of Goal Systems: Multifinality, Equifinality, and Counterfinality in Means—End Relations. In A. J. Elliot (Ed.), *Advances in Motivation Science* (Vol. 2, pp. 69-98). Elsevier. <https://doi.org/10.1016/bs.adms.2015.04.001>

Kruglanski, A. W., Shah, J. Y., Fishbach, A., Friedman, R., Woo Young, C., & Sleeth-Keppler, D. (2002). A theory of goal systems. In *Advances in Experimental Social Psychology* (Vol. 34, pp. 331-378). Academic Press. <https://doi.org/10.1016/S0065-2601(02)80008-9>

Latham, G. P., Ganegoda, D. B., & Locke, E. A. (2011). Goal-setting: A state theory, but related to traits. In *The Wiley-Blackwell handbook of individual differences.* (pp. 579-587). Wiley Blackwell. <https://doi.org/10.1002/9781444343120>

Miafodzyeva, S., & Brandt, N. (2013). Recycling Behaviour Among Householders: Synthesizing Determinants Via a Meta-analysis. *Waste and Biomass Valorization, 4*(2), 221-235. <https://doi.org/10.1007/s12649-012-9144-4>

Norton, T. A., Parker, S. L., Zacher, H., & Ashkanasy, N. M. (2015). Employee Green Behavior:A Theoretical Framework, Multilevel Review, and Future Research Agenda. *Organization & Environment, 28*(1), 103-125. <https://doi.org/10.1177/1086026615575773>

Osborne, J. (2013). *Best Practices in Data Cleaning: A Complete Guide to Everything You Need to Do Before and After Collecting Your Data*. Sage. <https://doi.org/10.4135/9781452269948>

Osborne, J. W. (2010). Data Cleaning Basics: Best Practices in Dealing with Extreme Scores. *Newborn and Infant Nursing Reviews, 10*(1), 37-43. <https://doi.org/https://doi.org/10.1053/j.nainr.2009.12.009>

Osborne, J. W., & Overbay, A. (2004). The power of outliers (and why researchers should always check for them). *Practical Assessment, Research, and Evaluation, 9*(1), 6.

Otto, S., Kibbe, A., Henn, L., Hentschke, L., & Kaiser, F. G. (2018). The economy of E-waste collection at the individual level: A practice oriented approach of categorizing determinants of E-waste collection into behavioral costs and motivation. *Journal of Cleaner Production, 204*, 33-40. <https://doi.org/10.1016/j.jclepro.2018.08.293>

Pallant, J. (2020). *SPSS survival manual: A step by step guide to data analysis using IBM SPSS* (7th ed.). Routledge.

Park, J., & Ha, S. (2014). Understanding consumer recycling behavior: Combining the theory of planned behavior and the norm activation model. *Family and Consumer Sciences Research Journal, 42*(3), 278-291. <https://doi.org/10.1111/fcsr.12061>

Parkinson, J., Russell-Bennett, R., & Previte, J. (2018). Challenging the planned behavior approach in social marketing: emotion and experience matter. *European Journal of Marketing, 52*(3/4), 837-865. <https://doi.org/10.1108/EJM-05-2016-0309>

Perugini, M., & Bagozzi, R. P. (2001). The role of desires and anticipated emotions in goal‐directed behaviours: Broadening and deepening the theory of planned behaviour. *British Journal of Social Psychology, 40*(1), 79-98. <https://doi.org/10.1348/014466601164704>

Qin, B., & Song, G. (2022). Internal Motivations, External Contexts, and Sustainable Consumption Behavior in China&mdash;Based on the TPB-ABC Integration Model. *Sustainability, 14*(13), 7677. <https://www.mdpi.com/2071-1050/14/13/7677>

Sykes, G. M., & Matza, D. (1957). Techniques of neutralization: A theory of delinquency. *American Sociological Review, 22*(6), 664-670. <https://doi.org/10.2307/2089195>

Tang, Z., Chen, X., & Luo, J. (2011). Determining socio-psychological drivers for rural household recycling behavior in developing countries: A case study from Wugan, Hunan, China. *Environment and Behavior, 43*(6), 848-877. <https://doi.org/10.1177/0013916510375681>

Triandis, H. C. (1977). *Interpersonal behavior*. Brooks/Cole Publishing Company.

Wang, Z., Guo, D., Wang, X., Zhang, B., & Wang, B. (2018). How does information publicity influence residents’ behaviour intentions around e-waste recycling? *Resources, Conservation and Recycling, 133*, 1-9. <https://doi.org/10.1016/j.resconrec.2018.01.014>

Xianfang, Z., Radford, S., & Mourali, M. (2017). Antecedents and Consequences of Environmental Beliefs: A Meta-Analysis. *AMA Marketing & Public Policy Academic Conference Proceedings, 27*, 55-56.

Zhang, S., Hu, D., Lin, T., Li, W., Zhao, R., Yang, H., Pei, Y., & Jiang, L. (2021). Determinants affecting residents’ waste classification intention and behavior: A study based on TPB and A-B-C methodology. *Journal of Environmental Management, 290*, 112591. <https://doi.org/10.1016/j.jenvman.2021.112591>

Zhang, Z. J., & Zhao, L. G. (2019). Voluntary monitoring of households in waste disposal: An application of the institutional analysis and development framework. *Resources, Conservation and Recycling, 143*, 45-59. <https://doi.org/10.1016/j.resconrec.2018.12.018>
